# Dentition and Its Derangements

**Published:** 1861-11

**Authors:** A. Jacobi


### Selections.

Dentition and its Derangements.—By *A. Jacobi, M. D.* —*Lecture V.*—That a large number of infants cut their teeth without any bad symptoms, has never been denied. Instead, however, of considering these cases as natural, they have been taken as exceptions; instead of looking for the causes of diseases in the age of the patient, and its various morbid dispositions in its constitution, or in direct injuries, both authors and the public have seemed to rest satisfied in the belief that the more an infant was disturbed with abnormal functions, the nearer came dentition to its natural standard. But all the symptoms observed during or before the protrusion of teeth do not come within the range of morbid affections; I have already spoken of some of the symptoms indicating the approximation of, or attending, the progress of dentition; I therefore shall not dwell upon them, but shall briefly enumerate such as are generally attributed to the protrusion of teeth. I may state that many explanations which would be here required, will naturally follow a physiological sketch of early infantile age, which I intend to give you in a future lecture.

The general irritability of the nervous system in teething children, is said to be increased. They are restless, sleepless, will suddenly awake from a short slumber, are peevish and cross, change their color frequently, and often urinate. I am certainly unwilling to deny the frequent occurrence of these symptoms in teething infants, but will take the liberty of stating, that in early infancy nervous symptoms are of frequent occurrence; that even the weight of the organs of the nervous system is greater in proportion than at any other period of human life, and its action may be supposed to be more powerful, and perhaps irregular; and that the very same symptoms attributed to teething are really being observed in almost all the affections occurring at this age. Both functional disorders and diseases are the more frequent the younger the individual; this is a fact clearly shown by all the statistics of both private and hospital practice, relating to both diseases, and the rate of mortality in this early period of life.

The local irritability produced by the process of dentition has often been noticed, and alluded to as a proof that there is something very peculiar and troublesome about this process. The infant is said to put its fingers into its mouth, and introduce anything it can lay hold on; it bites the nipples, and gentle rubbing of the gums causes an agreeable sensation. It is said to rub its lips, nose and eyes, and to move the occiput on the pillow, especially at the time of the protrusion of the incisors of the upper jaw.

Such are said to be the local symptoms of either approaching or present dentition; the latter term always being made use of to signify the final protrusion of the teeth. Is it remarkable that an infant will put its fingers into the mouth at this protracted period of teething, viz. from the fifth or sixth to the thirtieth month, when it has done so from birth? The very fact of the peculiar position of the fœtus in the uterus, the prominence of the action of the flexor over the extensor muscles, appear to be among the first causes of the new-born child's sucking its fingers. The great sensibility of the cutaneous nerves of the ends of the fingers and of the lips, which are moreover regularly exercised by the reception of food; the indistinct impression, in the infant, after having been nursed a number of times, of the lips and mouth being in some connection with the feeling of satisfaction, are the reasons why the infant sucks its fingers in the few weeks and months following its birth. Whichever explanation is correct, it is a fact that, from the hour of birth, the infant will either suck its fingers or keep them in the neighborhood of the mouth and nose. Nor is it astonishing that an infant will, during the time of dentition, take everything to its lips and into its mouth, after it has done so all its life.

The principal impressions an infant obtains depends on its relation to foods and drinks; eating is the only real propensity an infant has, and the mouth is known by experience to be the great receptacle destined for the reception of everything around; not to speak again of the lips being used as a means of touching, grasping, and learning the qualities of things. Everything living learns by experience and experiments, by physical impressions. All the sensory organs will be exercised for the purpose of understanding the impressions on the peripheric nerves, and the sensory organ first freely exercised by an infant is that of palpation. Further, a teething child will often bite the nipple, undoubtedly, but a number will not; and any child with any irritation of the cavity of the mouth, with any form of stomatitis, with any disease, in fact, which causes a sensation of uneasiness, will do the same. And finally, ought we to attribute the restless movements of the occiput on the pillow to teething, when every child affected with almost any affection of the brain, or its membranes, with hyperæmia of the cranial bones, with cutaneous eruptions of the cranial integuments, and rachitical affection of the bones, will be observed to do the same?

Why is it that the protrusion of the upper incisors is often attended with this restlessness, and almost regular moving to and fro of the head on the pillow? It means that irregularity, or anomalies in the protrusion of the upper teeth frequently depend on anomalous development of the upper jaw itself; and that the development of the upper jaw is generally in intimate connection with the development of the cranial bones. Thus you perceive, that when this is abnormal, and the upper jaw suffering accordingly in its general development, that often-mentioned symptom has nothing to do with the protrusion of teeth, as such, but must be referred to a defective or abnormal development of the cranial bones and subsequent anomalies in the structure or function of the brain. Instead, therefore, of pointing to dentition, especially to normal dentition, it indicates some more or less grave disturbance in the constitution or function of either the brain or its membranes, or its cranial or cutaneous integuments.

There are some objective symptoms announcing the approaching protrusion of teeth, which are of more or less importance. The gums will swell, and become looser and softer; or, which is more common, the alveolar margin will become thick, hard, flat, and prominent. This condition is always perceptible, and nevertheless we are liable to be mistaken as to its signification. I have seen a child, who had this prominence of the thickened alveolar margin over several teeth for a long time, without the teeth making their appearance. In fact, deep incisions had been made into the gum, more than two months before the first incisor cut through. This shows that although the normal process of dentition generally requires the condition of the alveolar margin described above, we are by no means justified in predicting a speedy protrusion of a tooth through the thickened and elevated wall. The prominence of the so-called dental cartilage is often said to be red, livid and soft. But in healthy infants, and with normal dentition, the contrary is generally the case. The mucous membrane of the mouth, although normal, is generally of a deeper color than the normal appearance of the gums, and frequently, in catarrhal affections of the mucous membrane of the mouth, the difference between its livid and softened velvet-like appearance, and the pale color and solid condition of the gums, is remarkable. Only when the protrusion of a tooth is very imminent, the gum will be in many cases a little sensitive, on being touched or pressed; and saliva and mucus are said to be secreted in a large quantity at the same time, until the gums become thinner and thinner, and the tooth protrudes.

Great importance is attached to salivation by the public, as a premonitory symptom of dentition; but it is a fact, that it will sometimes precede the breaking through of a tooth for a number of months, and will not cease after the tooth, or a group of teeth, have made their appearance. It is thought to be caused by the direct irritation of the gums, acting on the mucous membrane of the mouth, and the stenonian ducts and the salivary glands. It has even been considered to be the cause of a number of accidents occurring during dentition; the saliva and mucus were, in the opinion of a number of medical writers, swallowed, and proved to be the cause of vomiting and diarrhea, of erosions and aphthous inflammation of the mucous membrane. The truth is, that increased salivation is regularly observed in infantile age, long before, and during the first period of dentition. If, therefore, those authors were right, who believe it to depend on the irritation of the mucous membrane of the mouth, and of the salivary glands, there must be a constant irritation of the gums in every normal dentition. This, however, is not so, according to my preceding remarks. I will simply state now, that the increased secretion of mucus and saliva before this time does not depend on the protruding teeth, but is the result of the salivary glands and mucous follicles undergoing about this time a rapid process of development.

We shall have to return to this subject, and have to learn from a physiological sketch of the infantile organism, which I expect to give in this course of lectures, that a number of symptoms apparently affected by each other, and depending on each other, are but inordinate consequences of one and the same common cause. At all events, this will be readily understood by you, that the increased salivation need not be produced by some supposed constant irritation of the gums. At all events, you will not be deceived by the occasional emphatic statement of the following observations, which is meant to show that dentition in normal and robust children will be attended with copious salivation, while sickly and feeble children have no salivation to any amount. This appears to be true, but is not. The observation is imperfect in this, that healthy and robust children of four, six, or eight months, will generally, while awake, be in an upright position, thus dropping a large amount of the secreted mucus and saliva, and being constantly wet with it; while sickly and feeble children of the same age will, first, be a little backward in their general development, and moreover, have too little muscular power to allow them any but a supine position. Thus they will swallow most of the secretion, which more robust children will be constantly wet with.

I think it but reasonable to infer that if remarkable symptoms are the result of dentition, either normal or abnormal, a large number of anomalies must take place in the immediate neighborhood of the protruding tooth, if not in its own substance. Such affections are found, indeed, and known by the terms of *odontalgia*, *odontitis*, and *gingivitis*; but they are very rare affections, and the only idiopathic ones which are said to have occurred during, or rather in consequence of dentition.

Odontalgia, or neuralgia of the dental pulp, the dental nerve, is said to have been observed in teething children. What were the symptoms of this disease of dentition? Patient cried much, kept his fingers in the mouth, caught the breast greedily, and left off just as suddenly, was also constipated, but otherwise healthy, and there was but little injection, and intumescence of the gums. Exactly the same symptoms are reported to attend normal dentition, with the exception, perhaps, of constipation. But the restlessness of the infant was in connection with this constipation, and it screamed from colic pains. Although we are told by observers that the symptoms would disappear with the protrusion of the very first point of a tooth, the number of cases of this dental disease is so small, that we can not refrain from doubting the correctness of the diagnosis. In olden times, odontalgia from dentition has been observed a number of times; thus Karl Hindy has a chapter on the subject; but a more modern author, Hanmann, relates having seen two cases occurring during the protrusion of the molar teeth. Two cases in the lifetime of a medical man, who has met with many thousands of teething children; no pathognomic symptoms in these very cases to distinguish them from other complaints; no like observations in the practice of hundreds of other practitioners —all this looks rather suspicious, and leads us to infer, that this odontalgia depending on dentition, although its occasional occurrence during the protrusion of a tooth may have been observed, is rather doubtful.

Gingivitis, inflammatiun of the gums, is also reported to have been observed in the course of dentition. Its symptoms are the very same that have been given as premonitory of normal dentition, and in odontalgia, with the addition of intense injection, swelling, and heat of the gums and the mucous membrane of the mouth and pharynx. We are justified in doubting whether all these cases have been primary gingivitis, or whether or not the affections of the mouth and pharynx have been the primary diseases; the more so when we again are told of the presence of the very same symptoms as above, and moreover learn, that the gums will not only tolerate a moderate pressure while inflamed, but the patient feels relieved. That there can be a severe inflammation of the gums, in connection with the protrusion of a tooth, is proved by the difficulty sometimes, though rarely, met with by the protruding wisdom tooth, resulting from insufficient room, etc., but very rare it must be, as the termination in suppuration has been observed by but very few men, and but very seldom altogether. We are the more justified in so presuming, as we know of a number of cases of very severe and general stomatitis without the least affection of the gums, and of others where the gums were immensely swelled without injection, heat, or pain; and as the gums are generally very little apt to be affected by inflammatory action. Ulceration of the cheeks in the immediate neighborhood, or even anomalous protrusion of teeth, either deciduous or permanent, through the gums and alveolar process, in an oblique direction, are but seldom found to give rise to an inflammatory process in the gums.

Odontitis, or inflammation of the tooth, is the third local affection sometimes attributed to dentition. Again the same symptoms, pain, injection, swelling, are enumerated, and described as very intense and obstinate. Recovery would not always take place, although it would be the result, after days or weeks, in the majority of cases; but death would sometimes ensue under the symptoms of a thorough affection of the nervous system, or of a "typhoid fever." It would often be combined with other diseases, and, according to Schönbein, not unfrequently with rachitis. Jahn has made a number of *post-mortem* examinations in cases of odontitis, and what did he find in such children who died from inflammation of a tooth? Why, hyperæmia of the brain, acute hydrocephalus, "gastromalacia," and always violent inflammation of the gums and alveoli, with sometimes a dark bluish color of the alveolar margin. This latter shows certainly injection, but the former prove those children to have suffered from, and died of cerebral diseases. The connection of rachitis also points to the slight importance of the local affection, showing that the principal danger has been observed to be derived from constitutional or local ailings, not at all depending on or connected with, the local process of the protrusion of a tooth. I have to state, finally, that there is no such thing as odontitis proper, the dental tissue being too hard and deprived of vessels for an inflammatory process to take place. What has been called by this name, is either endodontitis, or perodontitis. The former is inflammation of the inner dental pulp, richly endowed with nerves and vessels, in which stasis and chemical changes may take place, and intense pain be felt, and central caries brought on. This form will sometimes be observed, but in advanced age, and not rarely in very robust and otherwise healthy men. Perodontitis is inflammation of the periosteum surrounding the root of a tooth, producing a heating pain, especially in the warm temperature of the bed. The tooth appears to be elongated, and feels sore on pressure, until either recovery has taken place or suppuration, which will permit the tooth to be removed without much difficulty. That the gums suffer simultaneously, is but natural. But this affection is also observed, almost exclusively, in adults. —*American Medical Times.*

